# Attenuation of acute lung injury in a rat model by *Semen Cassiae*

**DOI:** 10.1186/s12906-017-1747-7

**Published:** 2017-04-28

**Authors:** Xiuqing Chen, Xianming Zhang, Jie Zhang, Yang Gao, Zhaohui Yang, Shanshan Li, Haiwen Dai

**Affiliations:** 1 Department of Emergency, Zhejiang Hospital, 12 Lingyin Road, Hangzhou, 310013 China; 2 Department of Respiratory Medicine, First Affiliated Hospital of Guizhou Medical University, Guiyang, 550004 China; 3 Department of Respiratory Medicine, Henan Provincial People’s Hospital, 7 Weiwu Road, Zhengzhou, 450003 China; 4 Department of Respiratory Medicine, Beijing Anzhen Hospital of the Capital Medical University, 2 Anzhen Road, Beijing, 100029 China; 5 Department of Respiratory Medicine, the First People’s Hospital of Qingdao Economic & Technological Development Area, 9 Huangpujiang Road, Qingdao, 266000 China

**Keywords:** Acute lung injury, *Semen Cassiae*, Inflammation

## Abstract

**Background:**

Acute lung injury (ALI) is an inflammatory disorder. *Semen Cassiae* has potent anti-inflammatory activities. The aim of our study was to investigate whether *Semen Cassiae* plays a protective effect on lipopolysaccharide (LPS)-induced ALI and, if so, to elucidate its potential mechanism.

**Methods:**

Male Sprague–Dawley rat lungs were injured by intratracheal instillation of LPS. Rats were treated with *Semen Cassiae* or vehicle 3 h after LPS challenge. Samples were harvested 24 h post-LPS administration. We also investigated the effects of *Semen Cassiae* on LPS stimulation in RAW 264.7 cells.

**Results:**

LPS administration markedly induced pulmonary edema and polymorphonuclear neutrophil influxes. These changes were significantly attenuated in *Semen Cassiae* treated group. Moreover, *Semen Cassiae* markedly reduced pulmonary interleukin (IL)-6, tumor necrosis factor (TNF)-α, and 8-hydroxy-2′-deoxyguanosine (8-OHdG) levels. The pulmonary soluble epoxide hydrolase (sEH) activity and the DNA binding activity of Nuclear factor (NF)-κB were significantly inhibited in *Semen Cassiae* treated group. Furthermore, *Semen Cassiae* treatment significantly increased epoxyeicosatrienoic acids (EETs), and heme oxygenase-1 (HO-1) activity. Our in vitro study demonstrates that *Semen Cassiae* treatment may inhibit LPS induced IκBα phosphorylation and NF-κB p65 nucleus translocation.

**Conclusion:**

*Semen Cassiae* protects LPS-induced ALI in rats. *Semen Cassiae* can be developed as a novel treatment for ALI.

**Electronic supplementary material:**

The online version of this article (doi:10.1186/s12906-017-1747-7) contains supplementary material, which is available to authorized users.

## Background

Acute lung injury (ALI)/acute respiratory distress syndrome (ARDS) is severe disorder of lungs with high mortality [1, 2]. The underlying mechanisms of ALI/ARDS are complex. It is suggested that systemic inflammatory response and oxidative stress play an important role in the development of ALI/ARDS [3]. Thus, it is logically to hypothesize that inhibiting inflammation and oxidative stress are effective strategy to reduce ALI/ARDS.

A recent study showed that soluble epoxide hydrolase (sEH) plays a prevailing role in the pathogenesis of acute lung injury (ALI) [4]. sEH converted epoxyeicosatrienoic acids (EETs), an endothelium-derived factor with anti-inflammatory effects, into dihydroxyeicosatrienic acids (DHET) [5]. Inhibition of sEH reduced cisplatin-induced renal apoptosis [6]. sEH inhibition has also shown protective effect on cardiomyocyte via EETs-dependent mechanisms [7]. Moreover, a sEH inhibitor reduced kidney ischemia-reperfusion injury by modulating inflammatory mediators [8]. These evidences suggest that sEH is a vital pharmacologic target for inflammation. Thus, drugs which have sEH inhibiting features may exert anti-inflammatory action.

Heme oxygenase (HO)-1, a rate-limiting enzyme in oxidative degradation of heme to biliverdin, plays an important effect in maintaining oxidative/antioxidant homeostasis [9]. Elevating HO-1 activity may reduce ALI [10, 11]. It is now widely accepted that HO-1 contributes to the antioxidative defenses in ALI [10, 11].

*Semen Cassiae*, the seeds of *Cassia tora*, contains multiple active ingredients such as anthraquinone compounds, cassiaside, and sitosterol. *Semen Cassiae* is a traditional Chinese medicine that has been demonstrated with anti-inflammatory and antioxidant features [12, 13]. Recent in vitro study demonstrated that *Semen Cassiae* has inhibitory effect on sEH [14]. Cui et al. reported that *Semen Cassiae* may reduce hepatic injury in rats [12]. However, it is still unknown that whether *Semen Cassiae* has any protective effect on ALI. The present study was conducted to test the hypothesis that *Semen Cassiae* attenuates lipopolysaccharide (LPS)-induced ALI through inhibition of inflammation and oxidative stress.

## Methods

### Ethics statement

All animal experiments were performed in accordance with the guidelines from the Administration of Animal Experiments for Medical Research Purposes issued by the Ministry of Health of China. The protocol used was reviewed and approved by the Animal Experiment Administration Committee of Zhejiang Hospital (Hangzhou, China).

### Animals

Male Sprague–Dawley rats (160–180 g) were purchased from Zhejiang University (Hangzhou, China) and housed separately in a temperature controlled room on a 12 h light/12 h dark cycle. Animals received standard laboratory rodent chow ad libitum.

### Rats model of ALI

All procedures were performed under anesthesia. Animals were randomly divided into various groups: control, LPS + vehicle, LPS + *Semen Cassiae*. *Semen Cassiae* was purchased from Shunchang herb store (Shanghai, China) and authenticated by Zhejiang Institute of Drug Control (Hangzhou, China). We obtained a crude decoction of *Semen Cassiae* by boiling 50 g *Semen Cassiae* in 500 mL distilled water for 2 h. Then, the decoction was filtrated by a filter and concentrated to 50 mL by a low-pressure evaporator. A freeze-drying technique was used to process 5 g of *Semen Cassiae* powder through the resultant decoction. Before use, the *Semen Cassiae* powder was dissolved in phosphate buffered saline (PBS). In LPS + *Semen Cassiae* group, rats were fed once with 10 g of *Semen Cassiae* per kilogram of a rat 3 h after LPS administration. The dose of *Semen Cassiae* selected in our study was based on a previously published article [12]. Appropriate amount of the *Semen Cassiae* powder was dissolved in 1 mL PBS before use. Equivalent PBS was administrated for control group and LPS + vehicle group. Briefly, on the day of the experiment, animals were anesthetized by intraperitoneal injection of a cocktail of ketamine (100 mg/kg)/xylazine (5 mg/kg). Then, 50 μL of LPS (Sigma, St. Louis, MO, USA) intratracheal instillation was performed to induce ALI as described previously [15]. Before use, LPS was dissolved in sterilized PBS at 10 mg/mL. We administrated equivalent PBS for control group. Rats in each group were sacrificed at 24 h after LPS or PBS challenge by cervical dislocation and exsanguinated by cutting the vena cava inferior. We homogenized lung tissue samples in PBS on ice to make the 10% pulmonary homogenate and stored at −70 °C for further analysis.

### Pulmonary histological analysis

Pulmonary specimen was fixed with 10% buffered formalin and stained with hematoxylin and eosin (H&E). The degree of lung injury was scored by a histologist unaware of the sample groups. The degree of lung injury was scored according to: infiltration of neutrophils and thickness of the alveolar wall. Each assessment was graded as: 0, appears normal; 1, light; 2, moderate; 3, severe. We calculated a total score for each animal [16].

### The ratio of the lung wet to dry weight (W/D) analysis

The lung was isolated and weighed before being dried in an oven at 80 °C for 48 h. The dried lung was weighed again to calculate the pulmonary W/D ratio.

### Measurement of sEH activity and EETs levels

Pulmonary homogenate sEH activity and EETs levels were measured by an indirect method using a 14, 15-EET/DHET ELISA kit (Detroit R&D, Michigan, USA) as described previously [4].

### Measurement of pulmonary inflammatory mediators

Pulmonary homogenate interleukin (IL)-6 and tumor necrosis factor (TNF)-α were measured by use of a IL-6 or TNF-α ELISA kit (R&D Systems, Inc., USA) according to the manufacturers’ manual.

### Determination of oxidative stress and HO-1 activity

The level of 8-hydroxy-2′-deoxyguanosine (8-OHdG), an indicator of oxidative stress, was measured using ELISA kits (Biotechnology Co., Ltd. Shanghai, China) according to the manufacturers’ manual.

An indirect method was used to detect the pulmonary homogenate HO-1 activity by measuring the rate of appearance of bilirubin as described previously [17].

### Nuclear factor (NF)-κB activity assay

The DNA binding activity of NF-κB in the lung was determined by ELISA. An ELISA-based NF-κB p50/p65 transcription factor assay kit (Chemicon, Temecula, CA) was used in the current study according to the manufacturer’s instructions.

### Cell culture study and cell viability assay

We maintained RAW264.7 cells (the Cell Storehouse of the Chinese Academy of Science, Shanghai, China) in Dulbecco’s modified Eagle’s medium (DMEM) (Life Technologies, USA) at 37 °C under 5% CO_2_. The DMEM contains 10% heat-inactivated fetal bovine serum (FBS), 100 U/mL penicillin, and 100 μg/mL streptomycin.

3-(4, 5-dimethylthiazol-2-yl)-2, 5-diphenyltetrazolium bromide (MTT) (Sigma Chemical Co. St. Louis, MO, USA) assay was used to determine cell cytotoxicity that caused by *Semen Cassiae*. In brief, RAW 264.7 cells (5 × 10^4^ cells/well) were treated with various concentrations of *Semen Cassiae* (1 μmol/L, 10 μmol/L and 100 μmol/L) and incubated in 48-well plates for 24 h. Before use, the *Semen Cassiae* powder was dissolved in PBS. Equivalent PBS was administrated for control group. Then, 10 μL of MTT solution (5 mg/mL) was added to each well of cells and further incubated for 4 h. After the incubation, the supernatant was washed out. The insoluble formazan product was dissolved in 200 μL of dimethyl sulfoxide (DMSO) for 15 min, and the optical density (OD) at 570 nm was measured using a Microplate Reader. Cell viability in control medium was represented as 100%.

### Western blotting

The cells were pretreated with *Semen Cassiae* (1 μmol/L, and 10 μmol/L) for 1 h and then the cells were stimulated with LPS (1 μg/mL) or PBS for 20 h. Before use, the *Semen Cassiae* powder was dissolved in PBS. Equivalent PBS was administrated for control group and LPS + vehicle group. Nuclear and cytoplasmic protein from the supernatants was extracted for Western blot analysis using a Nuclear/Cytosol Extraction kit (BioVision, Inc., Mountain View, CA, USA). Rabbit NF-κB p65 and IκBα polyclonal antibodies (diluted 1:500; Santa Cruz Biotechnology; Santa Cruz, CA) were incubated with the blots overnight at 48 °C. The blots were washed in TBST buffer. Then, secondary antibody, horseradish peroxidase–linked anti–rabbit IgG (Cell Signaling Technology, Danvers, MA), were incubated with the blots for 1 h at room temperature. An anti-TATA antibody (diluted 1:1000; Abcam, Cambridge, MA, USA) and β-actin (diluted 1:200; Santa Cruz Biotechnology) were used as controls for nuclear and cytoplasmic proteins, respectively. We developed the blots by using an enhanced chemiluminescence detection kit (Amersham, Buckinghamshire, UK) and exposed on Hyperfilm ECL (Amersham, Buckinghamshire, UK). The NIH ImageJ software (National Institutes of Health, Bethesda, MD, USA) was used to quantify protein band concentrations.

### Statistical analysis

We presented all data as mean ± SEM. All data were analyzed with the SPSS 17.0 program (IBM, Armonk, USA). *P* < 0.05 was accepted as statistically significant. Differences between groups were carried out using one-way analysis of variance (ANOVA) followed by the Student-Newman-Keuls method. We performed histopathological scores by using Kruskal-Wallis one-way analysis of variance on ranks and the Student-Newman-Keuls method.

## Results

### Effects of *Semen Cassiae* on ALI

**Fig. 1:**
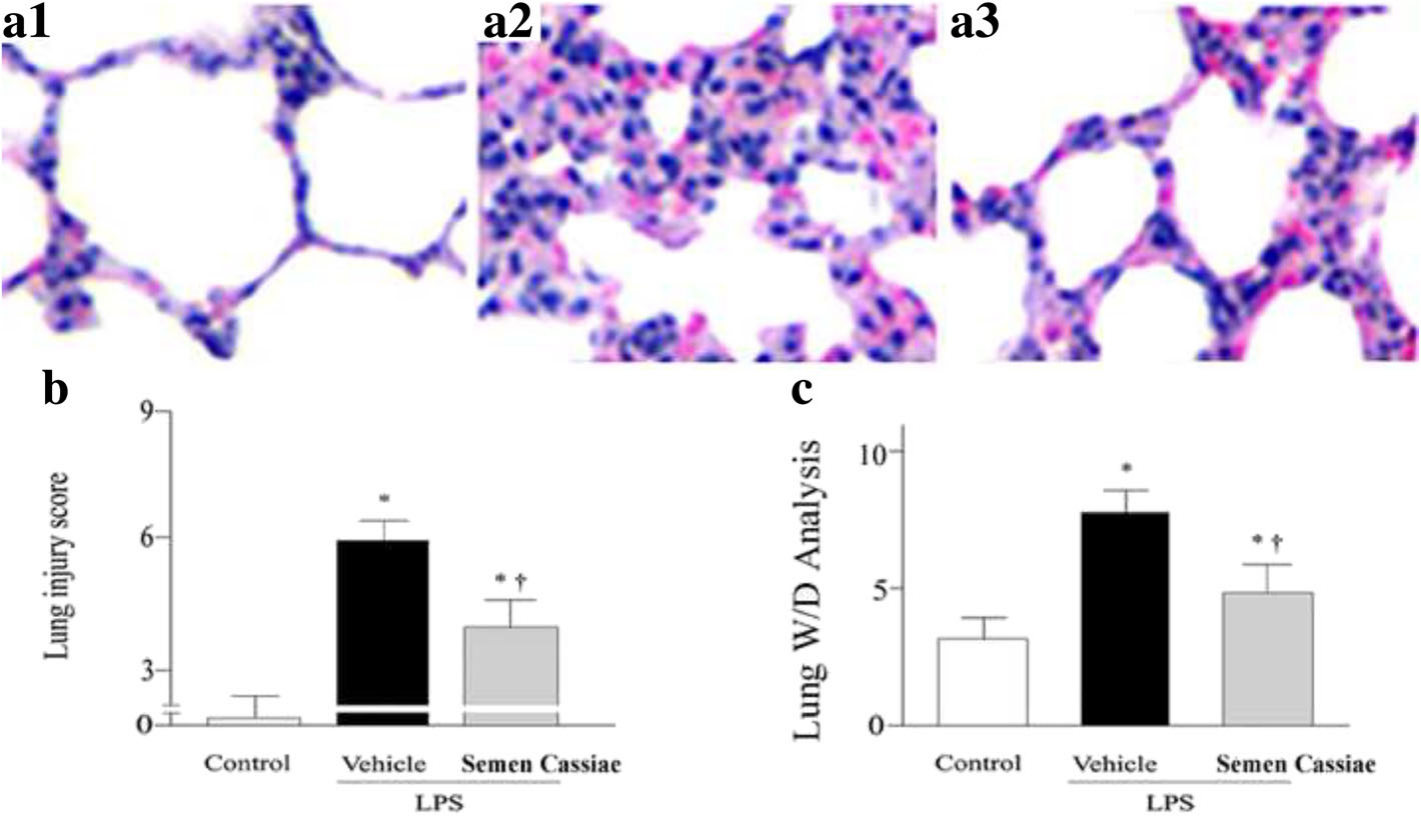
Morphologic alterations of the lung were determined by photomicrography. a1) Photomicrograph of a pulmonary section from a rat of control group. a2) Photomicrograph of a pulmonary section from a rat of lipopolysaccharide (LPS) + Vehicle group. a3) Photomicrograph of a pulmonary section from a rat of LPS + *Semen Cassiae* group. Magnification: ×400. Alterations of lung injury scores (b), or the ratio of the lung wet to dry weight (W/D) (c) in control or LPS challenged rats treated with vehicle or *Semen Cassiae*. Data are expressed as the mean ± SEM and compared by one-way analysis of variance and the Student-Newman-Keuls method. We performed histopathological scores by using Kruskal-Wallis one-way analysis of variance on ranks and the Student-Newman-Keuls method. ^*^ *P* < 0.05 when compared with control group; ^†^ *P* < 0.05 when compared with LPS + Vehicle group

As shown in Fig. 1, LPS challenge significantly induced lung tissue histopathological changes characterized by lung edema, destruction of pulmonary architecture, alveolar hemorrhage, and neutrophil infiltration (Fig. 1a2). These changes were markedly improved by *Semen Cassiae* treatment (Fig. 1a3). Moreover, as indicated in Fig. 1b, the lung injury score was significantly dampened in *Semen Cassiae* treated group. The lung wet to dry weight ration, an indicator of pulmonary edema, was markedly attenuated by *Semen Cassiae* treatment (Fig. 1c).

### Effects of *Semen Cassiae* on pulmonary inflammatory mediators

**Fig. 2:**
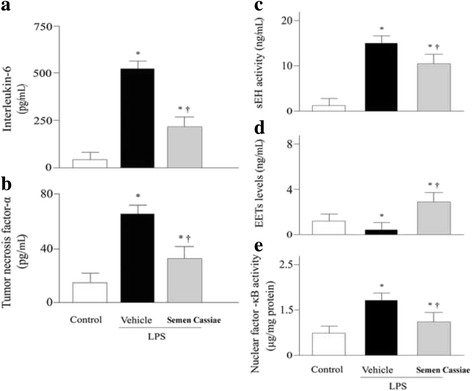
Alterations of interleukin (IL)-6 (**a**), tumor necrosis factor (TNF)-α (**b**), soluble epoxide hydrolase (sEH) (**c**), epoxyeicosatrienoic acids (EETs) (**d**), and nuclear factor (NF)-κB DNA binding activity (**e**) in control or lipopolysaccharide (LPS) challenged rats treated with vehicle or *Semen Cassiae*. Data are expressed as the mean ± SEM and compared by one-way analysis of variance and the Student-Newman-Keuls method. ^*^ *P* < 0.05 when compared with control group; ^†^ *P* < 0.05 when compared with LPS + Vehicle group

Pro-inflammatory cytokines play an important effect in the development of ALI/ARDS. In the present study, LPS challenge markedly increased pulmonary IL-6 and TNF-α level (Fig. 2a, and b). Animals treated with *Semen Cassiae* had significantly lower IL-6 and TNF-α levels compared with vehicle treated group (Fig. 2a, and b).

### Effects of *Semen Cassiae* on sEH activity and EETs levels

The activity of sEH was increased significantly in LPS + vehicle treated group compared with control (Fig. 2c). *Semen Cassiae* treatment markedly reduced the LPS-induced the elevation of sEH activity (Fig. 2c). EETs are known as an anti-inflammatory factor. In the present study, the EETs levels in pulmonary homogenate were significantly elevated in *Semen Cassiae* treated group compared with LPS + vehicle treated group (Fig. 2d).

### Effects of *Semen Cassiae* on 8-OHdG

**Fig. 3:**
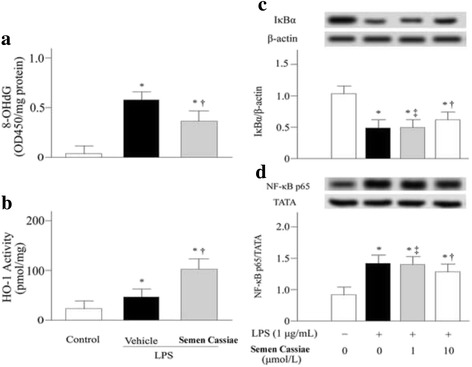
Alterations of 8-hydroxy-2′-deoxyguanosine (8-OHdG) (**a**), and heme oxygenase (HO)-1 activity (**b**) in control or lipopolysaccharide (LPS) challenged rats treated with vehicle or *Semen Cassiae*. Alterations of IκBα levels in cytoplasm (**c**), and Nuclear factor (NF)-κB p65 levels in nuclear (**d**) in RAW264.7 cells in control or lipopolysaccharide (LPS) challenged group pretreated with vehicle or *Semen Cassiae*. Data are expressed as the mean ± SEM and compared by one-way analysis of variance and the Student-Newman-Keuls method. ^*^ *P* < 0.05 when compared with control group; ^†^ *P* < 0.05 when compared with LPS + Vehicle group. ^‡^ *P* > 0.05 when compared with LPS + Vehicle group

Pulmonary concentrations of 8-OHdG were measured to investigate oxidative stress. 8-OHdG levels were markedly elevated in LPS + vehicle treated group (Fig. 3a). *Semen Cassiae* treatment significantly dampened 8-OHdG concentrations (Fig. 3a).

### Effects of *Semen Cassiae* on HO-1 activity

HO-1 has antioxidant features. Our results showed an increase of HO-1 activity in pulmonary homogenate in LPS + vehicle treated group (Fig. 3b). This result suggests an endogenous anti-oxidant mechanism. However, this protective effect was limited, as marked increased 8-OHdG levels were observed in the LPS + vehicle treated group (Fig. 3a). *Semen Cassiae* treatment significantly increased HO-1 activity compared with LPS + vehicle treated group (Fig. 3b).

### Effects of *Semen Cassiae* on cell viability

In order to test the cytotoxic effect of *Semen Cassiae*, the MTT assay was employed. Our results indicated that the viability of RAW 264.7 cells was not affected by *Semen Cassiae* treatment at concentrations up to 10 μmol/L (Additional file 1: Table S1).

### Effects of *Semen Cassiae* on NF-κB activation

Expressions of numerous proinflammatory cytokines were regulated by NF-κB which playing a vital role in the pathogenesis of ALI/ARDS [18–21]. We investigated the effect of *Semen Cassiae* on NF-κB activation in vivo and in vitro*.* Our Western blot analysis results indicated that the IκBα expression in the cytoplasm of RAW 264.7 cells was markedly decreased 20 h after LPS stimulation (Fig. 3c). Meanwhile, the NF-κB p65 in the nucleus of RAW 264.7 cells was significantly increased (Fig. 3d). *Semen Cassiae* inhibited the degradation of IκBα and prevented the translocation of NF-κB into the nucleus in LPS stimulated RAW 264.7 cells (Fig. 3c, and d). Moreover, the NF-κB DNA binding activity in lungs was markedly inhibited in *Semen Cassiae* treated group compared with vehicle group (Fig. 2e).

## Discussion

*Semen Cassiae* has anti-inflammatory and antioxidant features [12, 13]. However, the effect of *Semen Cassiae* on ALI, an inflammatory disorder, is still unclear. Our current study demonstrates the protective effect of *Semen Cassiae* on LPS-induced ALI characterized by reduced pulmonary edema and inflammation. The beneficial role of *Semen Cassiae* may be mediated via inhibiting sEH activity and NF-κB activation.

The current study indicates that *Semen Cassiae* treatment is associated with a decrease in sEH activity and elevating EETs levels. This result suggests a potential sEH inhibition activity of *Semen Cassiae* in vivo. Pharmacological inhibition of sEH has shown protective effect in different animal studies. Motoki et al. reported that sEH inhibition can reduce myocardial ischemia-reperfusion injury [22]. Cisplatin-induced renal apoptosis was attenuated by a sEH inhibitor via EETs dependent mechanisms [6]. Moreover, LPS-induced ALI was dampened by a sEH inhibitor in a preclinical research [4]. In the present study, pulmonary inflammation and injury was significantly reduced in *Semen Cassiae* treated group. This result indicated a protective effect of *Semen Cassiae* on ALI. The benefit role of *Semen Cassiae* may be associated with sEH inhibition. It has been reported that the protective effect of sEH inhibition may be mediated through EETs [6]. Mitochondrial function of cardiomyocytes exposed to LPS was significantly improved by 14, 15-EET in an in vitro study [23]. And LPS-induced cardiomyocytes cytotoxicity can be reduced by 14, 15-EET through peroxisome proliferator-activated receptors γ (PPARγ) signaling pathway [23]. Evidence has shown that protective effects of EETs on cerebral I/R injury is associated with phosphatidylinositol 3-kinase (PI3K)/Akt pathway and ATP-sensitive potassium (KATP) channels [24]. In our study, the EETs level was significantly increased in *Semen Cassiae* treated group. The increased EETs level may contribute to the protective effect of *Semen Cassiae* on ALI.

HO-1 is an inducible form of HO which plays an important role on reduction of reactive oxygen species (ROS) [25]. As oxidative stress is thought to play a vital effect in the pathogenesis of ALI [3], drugs have anti-oxidant features may play a protective effect on ALI. In the present study, HO-1 activity was significantly elevated in *Semen Cassiae* treated group. Moreover, the 8-OHdG levels, an indicator of oxidative stress, were markedly reduced in *Semen Cassiae* treated group. These results indicated an anti-oxidant action of *Semen Cassiae*. Previous studies have shown the anti-oxidant features of *Semen Cassiae* [13]. However, its potential mechanisms are still unclear. Studies have indicated that the expression of HO-1 protein and HO-1 activity could be induced by EETs [18]. In the current study, the EETs level was markedly increased in *Semen Cassiae* treated group. Thus, it seems reasonable to propose that *Semen Cassiae* increases HO-1 activity through inhibiting sEH and elevating EETs.

NF-κB, a well-documented transcription factor, regulates expressions of numerous proinflammatory cytokines such as TNF-α, and IL-6 [23]. It has been reported that NF-κB activity is increased in patients with ARDS [20, 21]. In animal studies, inhibition of NF-κB activation can dampen ALI [26, 27]. Evidence has shown that following LPS stimulation, NF-κB was activated [19]. In the current study, we examined the effect of *Semen Cassiae* on NF-κB activation. Our Western blot analysis results shown that the degradation of IκBα and the translocation of NF-κB p65 into the nucleus were markedly inhibited in *Semen Cassiae* pretreated RAW 264.7 cells. As Western blot analysis can not detect the activity of NF-κB, we performed an ELISA-based method to detect the NF-κB DNA binding activity. In our animal studies, the NF-κB activity in pulmonary homogenate was significantly attenuated by *Semen Cassiae* treatment. Consistent with previous findings [26], inhibition of NF-κB was followed by decreased proinflammatory cytokines expression. NF-κB has been suggested as a key pharmacologic target for ALI [19]. The NF-κB inhibiting effect of *Semen Cassiae* may, in part, contribute to the reduced ALI in rats.

## Conclusions

*Semen Cassiae* protects LPS-induced ALI in rats. *Semen Cassiae* can be developed as a novel treatment for ALI.

## Additional file

Additional file 1: Table S1. Cell viability assay measured by 3-(4, 5-dimethylthiazol-2-yl)-2, 5-diphenyltetrazolium bromide (MTT) (DOC 25 kb)
