# Supplementary material for: Attenuation of acute lung injury in a rat model by Semen Cassiae
Source: BMC Complement Altern Med. 2017 Apr 28;17:234. doi: 10.1186/s12906-017-1747-7 (PMC5408400; doi:10.1186/s12906-017-1747-7)
Supplement: Additional file 1: Table S1. — Cell viability assay measured by 3-(4, 5-dimethylthiazol-2-yl)-2, 5-diphenyltetrazolium bromide (MTT) (DOC 25 kb) [file 12906_2017_1747_MOESM1_ESM.doc]

**Table S1. Cell viability assay measured by 3-(4, 5-dimethylthiazol-2-yl)-2, 5-diphenyltetrazolium bromide (MTT)**

| **Group Control *Semen Cassiae* (μmol/L)**  1 10 100 |
| --- |
| **Cell viability (%)** 100 100^#^ 99^#^ 78*^*^* |

^#^P>0.05 when compared with control group; *^*^* P < 0.05 when compared with control group.

Equivalent phosphate buffered saline (PBS) was administrated for control group.
